# Miscellany

**Published:** 1910-06

**Authors:** 


### MISCELLANY.

THE United States Civil Service Commission announces an examination on June 15, 1910, at the usual places, to secure eligibles from which to make certification to fill at least two vacancies in the position of medical interne (male), Government Hospital for the Insane, Washington, D. C., at $600 per annum each, with maintenance, and vacancies requiring similar qualifications as they may occur in that hospital, unless it shall be decided in the interests of the service to fill either or both of the vacancies by reinstatement, transfer, or promotion. From the grade of medical interne the hospital makes promotions to the higher positions in the medical staff as vacancies occur.

As considerable difficulty has been experienced in filling vacancies in the position of medical interne in the Hospital Service during the past several years, owing to the limited number of eligibles available, qualified persons are urged to enter this examination.

Applicants should at once apply either to the United States Civil Service Commission, Washington, D. C., or to the secretary of the board of examiners at any place mentioned in the list printed hereon, for application Form 1312. No application will be accepted unless properly executed and filed with the Commission at Washington. In applying for this examination the exact title, as given at the head of this announcement, should be used in the application.

---

Dr. Murray Galt Motter, secretary, announces that it will be possible for the medical and pharmaceutical press and the organisations represented in the Pharmacopeial Convention of 1910, to secure, at a cost of five cents per folio of one hundred words, a carbon copy of the stenographic report of the proceedings of the convention. Arrangements should be made promptly, with Dr. Motter, Washington, D. C., and applications must be accompanied by a certified check for one hundred dollars as a guarantee of the payment of the final cost of the work.

---

The United States Civil Service Commission announces an examination on June 8, 1910, to secure eligibles from which to make certification to fill a vacancy in the position of bacteriologist and pathologist in the Bureau of Science, Manila, P. I., at a salary of $2,250 per annum, and a vacancy in the position of assistant professor of bacteriology and pathology in the Philippine Medical School at a salary of $2,000 per annum, and vacancies requiring similar qualifications as they may occur in the Philippine service.

It will not be necessary for applicants to appear at any place for examination. Their eligiblity will be determined upon the evidence furnished in connection with application and examination Form 375 concerning their education, training, and experience.

Applicants should at once apply to the United States Civil Service Commission, Washington, D. C., for application Forms 2 and 375. No application will be accepted unless properly executed and filed, in complete form, with the Commission at Washington prior to the hour of closing business on June 8, 1910.

